# Vaccines Against Human Papilloma Virus and Cervical Cancer: An Overview

**DOI:** 10.4103/0970-0218.42045

**Published:** 2008-07

**Authors:** Savita Sharma

**Affiliations:** Department of Community Medicine, Indira Gandhi Medical College, Shimla - 171 001 (HP), India

**Keywords:** Human papilloma virus, vaccine

## Abstract

The paradigm of preventing human papilloma virus (HPV) infection through currently approved vaccines, namely, Gardasil, manufactured by Merck and Co., Inc. (Whitehouse Station, NJ) and Cervarix, manufactured by GlaxoSmithKline (GSK, Philadelphia) holds tremendous promise for the developing countries in decreasing the burden of HPV infection and its sequelae, such as cervical cancer, genital warts and anogenital cancers. Effective screening programs that have reduced the burden of this killer disease in the developed countries are still lacking in India, despite the high incidence of cervical cancer and the implementation of the National Cancer Control Programme since 1975. The recent breakthrough in the global war against cervical cancer will provide new insight for meeting the future challenge of the prevention of cervical cancer in India.

## Introduction

Cervical cancer one of the leading cancers among women, which affects approximately 490,000 women each year, resulting in approximately 270,000 deaths worldwide.(1) Approximately 85% of women who die from cervical cancer belong to developing countries. According to the National Cancer Registry Programme of India, cancers of the uterine cervix and breast are the leading malignancies noted in Indian women.(2) It has been estimated that in India, 100,000 new cases of cervical cancer occur annually, and 70% or more of these cancers are stage III or higher at the time of diagnosis.(3) Lack of uniform policies and programs in organizing cervical cancer control activities no doubt have led to this disease remaining a major cause of death among Indian women. Clinical, molecular, and epidemiological investigations have shown strong etiological association between human papilloma virus (HPV) and cervical cancer.(4–6) In fact, almost all cervical cancers (99%) contain the genes of high-risk HPVs, most commonly types 16, 18, 31 and 45.(4,7)

This article reviews the available information on prophylactic vaccines against HPV infection and their relevance in the Indian scenario.

## HPV

HPVs belong to the family papillomaviridae. The genome of HPV consists of small (8-kb pairs) double-stranded circular DNA viruses that infect squamous epithelium of the genital tract, anal and perianal regions and mucosal epithelium of the larynx.(7) Low-risk HPVs such as HPV-6 and HPV-11 cause benign genital warts; on the other hand, high-risk types such as HPV-16 and HPV-18 are associated with the development of high-grade squamous intraepithelial lesions and cervical cancer.(7,8) It is estimated that HPV-16 accounts for approximately 60% of cervical cancers, with HPV-18 adding another 10%–20%. Other high-risk types include types 31, 33, 35, 39, 45, 51, 52, 56, 58, 59, 68, 73 and 82.(7,8) Results of various studies have demonstrated the prevalence of HPV genotypes in Indian women and the most common genotypes are HPV types 16 and 18.(9–11)

Although HPV-related cancers are found to be more common among women, increasing numbers of HPV-related carcinomas of the anal mucosa are also being reported in men who have sexual intercourse with men.(5) At birth, HPV can infect the mucosa of the pharynx and cause large wart-like lesions that can obstruct the respiratory passage.(7)

The pathogenesis of cervical cancer is initiated by HPV infection of the cervical epithelium during sexual intercourse. The initial morphological changes in the cervical epithelia, pathologically classified as cervical intraepithelial neoplasia (CIN1), are associated with continuous viral replication and virus shedding. In most infected women, the infection resolves spontaneously in less than two years; however, 3–10% of women, in whom these infections do not resolve, become persistent HPV carriers and constitutes a high-risk group in whom these infections progress to the cancer of the cervix.(12)

## Vaccines

Two distinct types of vaccines, one manufactured by Merck and Co., Inc. (Whitehouse Station, NJ), namely, Gardasil, and the other manufactured by GlaxoSmithKline (GSK) (Philadelphia), namely, Cervarix, are available for the prevention of the HPV infection.(13–17) Each vaccine includes HPV types 16 and 18, which account for approximately 70% of all cervical cancers worldwide. The Merck vaccine Gardasil in addition incorporates HPV types 6 and 11, associated with approximately 90% of genital warts.

In June 2006, the U.S. Food and Drug Administration (FDA) has approved a quadrivalent HPV recombinant vaccine developed by Merck and marketed under the trade name Gardasil, and in September 2006, it was recommended for immunization of girls of age 9–26 years and women. It is the first and only vaccine to prevent cervical cancer, vulvar and vaginal precancers caused by HPV types 16 and 18 and genital warts caused by HPV types 6 and 11. In July 2007, the European Committee for Human Medicinal Products recommended GlaxoSmithKline's experimental HPV vaccine Cervarix for sale and marketing in the European Union and will soon receive the final regulatory approval.

With regard to efficacy, both the vaccines have demonstrated remarkably similar results in the clinical trials and offered 100% protection for the type-specific lesions.(17,18)

The HPV vaccine is licensed for use among women and girls in the age group of 9–26 years. Ideally, females should be vaccinated before their sexual debut because the vaccine is most effective in women who have not yet acquired any HPV type infection. Vaccine schedule requires three doses to be administered over a period of six months (Gardasil: 0, 2 and 6 months and Cervarix: 0, 1 and 6 months). The dose of the quadrivalent vaccine is 0.5 ml to be administered intramuscularly in the deltoid muscle. The vaccine should be shaken well before use, stored at 2°C–8°C and should not be frozen. The price of three dose series of Merck's quadrivalent vaccine is approximately $360 in the US. Undoubtedly, the price will be one of the greatest barriers to the introduction of this vaccine in the developing countries. Drastic revision of the price will be required to introduce this vaccine in the poorest parts of the world to facilitate its timely utilization.

This will only be possible with subsidies provided from the Global Alliance for Vaccines and Immunization (GAVI) and assistance from agencies such as the World Health Organization (WHO), the Alliance for Cervical Cancer Prevention (ACCP), the International Agency for Research on Cancer (IARC), the Bill and Melinda Gates foundation and Program for Appropriate Technology in Health (PATH), who are ready to provide technical and financial support to developing countries; thus, this vaccine can be brought to India.

The decision of the Indian Government to join the clinical trials of the HPV vaccine holds some promise for the women to fight this battle against the malignant HPV infection. The ICMR signed an agreement with Merck and Co., and clinical trials for HPV vaccine are under study to observe its efficacy and safety in the Indian population. The Institute for Cytology and Preventive Oncology (ICPO) has signed a Memorandum of Understanding (MOU) with Merck and has taken the initiative to develop the vaccine against HPV.

## Approach and conclusion

A big challenge for us is to determine how to include HPV vaccine in a Cervical Cancer Control Programme, or what should be the mechanism to deliver the vaccine in Indian scenario?

Many critical questions will be raised by the clinicians, public health professionals and public at large, and these will have to be addressed before the vaccine is made available in India.

A major decisive question to be addressed is that how will the vaccine affect the tests used to screen Cervical Cancer? Will it be a replacement for the standard screening tests?

We all know well from our past experience that large-scale routine screening programmes, followed by treatment of the affected is difficult to achieve in our country; however, a cost-effective vaccine can act as another line of defense, provided the women who need it the most may be able to receive it. Unfortunately, the number of women receiving three or more antenatal checkups is quite disappointing (16.9% in Bihar and 26.3% in Uttar Pradesh) in some of the Empowered Action Group (EAG) states according to the National Family Health Survey (NFHS III);(18) this will prove to be a red herring to regard HPV vaccine as the ultimate approach toward the control of cervical cancer in India where a huge population lives below poverty line and the awareness among women about the control and treatment cervical cancer is very limited.

Moreover, the currently available HPV vaccines do not protect against all types of HPV; they provide protection against only the two most common high-risk types of HPV (16 and 18) which cause approximately 70% of cervical cancers. Females who are vaccinated could subsequently be infected with one of the less common types of HPV that the vaccine does not protect against. Furthermore, it is possible that sexually active women before vaccination could have been infected with a vaccine type HPV before vaccination.

Another tricky question is that who is eligible to be vaccinated primarily: women or girls?

This question needs to be answered in a very subtle manner because of religious and cultural taboos in our country.

Therefore, the core part of a good public health policy against cervical cancer should be vaccination and screening program complementing each other. The availability of cervical cancer vaccine will enable to reduce the cost burden for cervical cancer screening programs. It is time that the government takes steps to place cervical cancer screening, HPV vaccination and Information Education and Communication (IEC) for cancer control on its high agenda to reduce the increased burden of this disease and its consequences.
